# microRNA Response to *Listeria monocytogenes* Infection in Epithelial Cells

**DOI:** 10.3390/ijms13011173

**Published:** 2012-01-20

**Authors:** Benjamin Izar, Gopala Krishna Mannala, Mobarak Abu Mraheil, Trinad Chakraborty, Torsten Hain

**Affiliations:** 1 Institute of Medical Microbiology, Justus-Liebig-University, Frankfurter Strasse 107, Giessen D-35392, Germany; E-Mails: bizar@partners.org (B.I.); Gopala.K.Mannala@mikrobio.med.uni-giessen.de (G.K.M.); Mobarak.Mraheil@mikrobio.med.uni-giessen.de (M.A.M.); Trinad.Chakraborty@mikrobio.med.uni-giessen.de (T.C.); 2 Department of Internal Medicine, Massachusetts General Hospital, 55 Fruit Street, Boston, MA 02118, USA

**Keywords:** *Listeria monocytogenes*, microRNA, non-coding RNA, infection, epithelial cells, Caco-2

## Abstract

microRNAs represent a family of very small non-coding RNAs that control several physiologic and pathologic processes, including host immune response and cancer by antagonizing a number of target mRNAs. There is limited knowledge about cell expression and the regulatory role of microRNAs following bacterial infections. We investigated whether infection with a Gram-positive bacterium leads to altered expression of microRNAs involved in the host cell response in epithelial cells. Caco-2 cells were infected with *Listeria monocytogenes* EGD-e, a mutant strain (Δ*inl*AB or Δ*hly*) or incubated with purified listeriolysin (LLO). Total RNA was isolated and microRNA and target gene expression was compared to the expression in non-infected cells using microRNA microarrays and qRT-PCR. We identified and validated five microRNAs (miR- 146b, miR-16, let-7a1, miR-145 and miR-155) that were significantly deregulated following listerial infection. We show that expression patterns of particular microRNAs strongly depend on pathogen localization and the presence of bacterial effector proteins. Strikingly, miR-155 which was shown to have an important role in inflammatory responses during infection was induced by wild-type bacteria, by LLO-deficient bacteria and following incubation with purified LLO. It was downregulated following Δ*inl*AB infection indicating a new potent role for internalins in listerial pathogenicity and miRNA regulation. Concurrently, we observed differences in target transcript expression of the investigated miRNAs. We provide first evidence that *L. monocytogenes* infection leads to deregulation of a set of microRNAs with important roles in host response. Distinct microRNA expression depends on both LLO and pathogen localization.

## 1. Introduction

microRNAs (miRNAs) represent a class of small non-coding RNAs of ~22 nucleotides in length that repress gene expression on a post-transcriptional level by targeting the 3′ UTRs of cellular mRNA leading to its degradation or inhibition of translation [1]. miRNAs were implicated in a wide range of physiological as well as pathological processes, including inflammatory response, apoptosis, growth and cancer, neurodegenerative and cardiovascular diseases [2]. Increasing evidence suggests an important role of miRNAs in the immune response against infectious agents [3–5]. Previous work focused on and revealed direct anti-viral activity of miRNAs through repression of viral mRNA production [6]. Conversely, viral miRNAs were found to antagonize the host mRNA leading to a suppression of the anti-viral response [7].

Recently, a role of miRNAs in the response against bacterial pathogens has been proposed. miRNAs were shown to be effective against *Pseudomonas syringae* infection in plants [8]. Similar to viruses, *P. syringae* was found to secrete proteins that bind host miRNA and subsequently modulate immune response [8]. Furthermore, Rao and colleagues described the presence miRNAs expressed by pathogenic *Pseudomonas aeruginosa* strains which were isolated from adult patients with cystic fibrosis [9]. Xiao *et al.* uncovered a *Helicobacter pylori*-dependent induction of miR-146b and miR-155 in gastric epithelial cells with subsequent inhibition of IL-8, a central cytokine in the chemotaxis of leukocytes [10]. Further investigation revealed that miRNAs control major inflammatory pathways, such as the TLR-mediated activation of the NF-kB pathway [10]. While *P. syringae* and *H. pylori* remain extracellular during infection, a recent study showed altered immune response of mice deficient in miR-155 to the facultative intracellular pathogen *Salmonella* [5]. Schulte *et al.* uncovered the regulation of IL-6 and IL-10 by miRNAs of the let-7 family and miR-155 induction by secreted effector proteins of *Salmonella* rather than the invading pathogen [5].

In this study, we observed differential regulation of miRNAs and associated target transcripts in epithelial cells following infection with *Listeria monocytogenes. L. monocytogenes* is a Gram-positive, facultative intracellular bacterium that has been used widely for the elucidation of immune processes in a variety of hosts and tissues. *L. monocytogenes* facilitates its entry into non-phagocytic cells, such as epithelial Caco-2 cells, via surface bound and secreted effector proteins known as internalins. Internalized *Listeriae* are able to escape from the hostile phagocytic vacuole using the effector protein listeriolysin (LLO), a secreted toxin that is essential for the pathophysiology and intracellular survival of *L. monocytogenes*.

Using defined mutants that variously lack individual virulence factors, this study provides evidence that the ability and extent of *Listeria* induced regulation of host miRNAs strongly depends on cellular localization, on secreted and membrane-bound proteins of the pathogen.

## 2. Materials and Methods

### 2.1. Bacterial Strains and Growth Conditions

*L. monocytogenes* EGD-e [11] and its isogenic deletion mutants Δ*hly* [12] and Δ*inl*AB [13] were used in this study. Bacteria were grown in BHI broth overnight at 37 °C with shaking at 180 rpm. Overnight cultures were diluted into 1:50, grown to mid-exponential phase (OD_600nm_ = 1.0) and used for further experiments.

### 2.2. Eukaryotic Cell Culture

Human epithelial cells (Caco-2) were cultured in MEM with 10% fetal calf serum (FCS) and 5% non-essential amino acids, respectively. Cells were maintained at 37 °C in 5% CO_2_.

### 2.3. LLO Purification

LLO is expressed and purified from a recombinant *L. innocua* 6a strain harboring the *hly* gene [14]. Briefly supernatant fluids were concentrated using a Millipore filtration apparatus followed by batch absorption onto Q-sepharose (Pharmacia, Freiburg, Germany) and pre-equilibrated with loading buffer (50 mM NaH_2_PO_4_, pH 6.2). The non-absorbed fraction was centrifuged and desalted by transferring through a super loop to a HiPrep 26/10 desalting column (Pharmacia, Freiburg, Germany) where loading buffer (50 mM NaH_2_PO_4_, pH 6.2) was used to elute the desalted fraction. This fraction was subsequently filtered through a Millipore filter (0.22 μm) and loaded onto a Resource-S column previously equilibrated with 50 mM NaH_2_PO_4_, pH 6.2. The pure toxin eluted reproducibly from the column at 0.21 to 0.28 M NaCl using elution buffer (50 mM NaH_2_PO_4_ 1M NaCl, pH 5.6). Fractions were collected and individually tested for hemolytic activity. Yields of the toxins range from 1 to 5 mg/L supernatant with a hemolytic activity (HU) of 20,000 HU/mg purified protein. One hemolytic unit (HU) is expressed as the amount of toxin required to lyse 50% of a 1% suspension of sheep erythrocytes. The toxin showed a high purity as seen using SDS-PAGE analysis, was efficiently recognized with LLO-specific antibodies, and exhibited hemolytic activity on sheep erythrocytes at both pH 6.0 and pH 7.4 respectively.

### 2.4. Infection Assays and LLO Treatment

Caco-2 cells were maintained in 6-well plates following at conditions described above. Bacteria at MOI 10 were added to the monolayer of cells. One hour of post infection, followed by washing with 1 × PBS, cells were supplemented with fresh media containing 20 μg/mL gentamycin to remove extracellular bacteria. After one hour of gentamycin treatment cells were lysed using a mixture of RLT lysis buffer and 1% β-mercaptoethanol and used for RNA isolation.

LLO at different concentrations (25 ng/mL and 50 ng/mL), was preactivated with dithiothreitol before administration to Caco-2 cells. Following incubation with LLO for one hour, cells were lysed with RLT lysis buffer and 1% β-mercaptoethanol.

### 2.5. RNA Isolation

RNA was isolated from cell lysate samples using the Qiagen miRNeasy Kit. Briefly, cell lysate samples were transferred to the QIA Shredder column and centrifuged at 13,200 rpm. An equal amount of 70% of ethanol was added to the eluted sample and mixed thoroughly. These samples were passed through a nucleic acid binding column which is supplied by the miRNeasy Kit (Qiagen). The DNA present on the column was digested using RNase-free DNase (Qiagen) for 30 min at RT and RNA was eluted by RNase free water. The quantity of isolated RNA was measured with NanoDrop analyzer (NanoDrop Technology, Rockland, MA, USA) and quality was assessed by running the samples on Nano-chips for 2100 Bioanalyzer (Agilent, Böblingen, Germany).

### 2.6. miRNA Microarray

For this analysis we used the biochip “Geniom Biochip MPEA homo sapiens & mus musculus” (febit, Heidelberg, Germany). The probes are designed as the reverse complements of all major mature miRNAs and the mature sequences as published in the current Sanger miRBase release (version 14.0 September 2009, see http://microrna.sanger.ac.uk/sequences/index.shtml) for homo sapiens & mus musculus. Techniqual and procedural details are described in detail in supplementary material.

### 2.7. Reverse Transcription Reaction and Quantitative Real-Time PCR Analysis

First strand cDNA was generated for mRNA by using SuperScript II reverse transcriptase (Invitrogen) and miScript reverse transcription kit (Qiagen) for miRNAs using 1 μg of RNA for each reaction.

Quantitative real-time PCR analysis was performed by using AB Prism 7900 HT system. All forward and reverse primers used for PCR were purchased from Qiagen. We used RNUA1 as internal controls for miRNA expression normalization and HPRT for target mRNA expression normalization.

The reaction mixture volume of 25 μL for mRNA quantitative real-time PCR was applied using 100 ng cDNA for each reaction. For miRNA quantitative real-time PCR analysis 3 ng of cDNA per 50 μL reaction set-up was used. For each primer the efficiency was calculated by standard curve which was generated by using different concentrations of genomic DNA in real time PCR. The expression level of mRNA and miRNA was calculated by normalizing its quantity to the respective expression of the internal control in Caco-2 epithelial cells. Threshold cycle values (CT) of the tested transcripts were determined and normalized expression of each target gene was given as the ΔCT between the log2 transformed CT of the target gene and the log2 transformed CT of the internal control. Log2 transformed gene expression levels (ΔCT) of each target transcript were expressed as log2 differences from control (=log2 ΔΔCT method). Data was acquired and analyzed with the SDS 2.3 and RQ-Manager 1.2, respectively.

### 2.8. Statistical Data Analysis of Infection Experiments

All infection and toxin experiments were performed for a minimum of three times. Significant differences between two values were compared with a paired Student’s *t-*test. Values were considered significantly different when the *p* value < 0.05.

## 3. Results

### 3.1. *L. monocytogenes* Differentially Induces miRNAs Dependent on Cellular and Subcellular Localization

Based on miRNA expression analysis using microarrays, we selected a subset of miRNA candidates that were differentially deregulated following wild type infection of epithelial Caco-2 cells. We focused on miRNAs that have a biologically validated role *in vitro* or *in vivo*. These miRNAs were validated using qRT-PCR. Relative expression levels obtained by both techniques showed a robust correlation (Figure S1).

In addition to the wild-type infection, Caco-2 cells were infected with two isogenic mutant strains or incubated with purified listeriolysin (LLO). The *hly* mutant strain is unable to produce LLO and remains in the phagocytic vacuole after host cell infection. Δ*inl*AB remains in the extracellular space because of the inability to induce bacterial uptake into epithelial cells.

**Figure 1:**
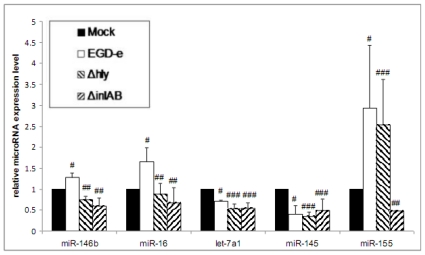
Measurement of miRNA candidates in infected Caco-2 cells compared to uninfected Caco-2 cells at 1 h post infection with *L. monocytogenes* EGD-e wild-type, Δ*hly* or Δ*inl*AB. Error bars indicate standard deviations. # significant difference compared to control (*p*-value < 0.05). ## significant difference compared with wild-type infection (*p*-value < 0.05), ### no significant difference compared with wild-type infection (*p*-value > 0.05).

Infection with wild-type bacteria leads to significantly increased expression of miR-146b, miR-16 and miR-155 expression in Caco-2 cells compared to non-infected cells (Figure 1).

As previously described for *Salmonella* [5], we also observed a significant downregulation of let-7a1 (Figure 1), a member of the let-7 family that is implicated in immune response and cancer development. We further observed a strong downregulation of miR-145 (Figure 1). A recent study demonstrated that blocking miR-145 led to a strong anti-inflammatory and reduced airway hyper responsiveness comparable to the effects obtained following glucocorticoid treatment [15].

Compared to wild-type infection both mutant strains induced significant deregulation of miR-146b and miR-16 (Figure 1). The expression differed with respect to the directionality of regulation for these miRNAs; while upregulated following wild-type infection, the expression of both miRNAs was decreased following infection with both mutant strains. Furthermore, we observed significant downregulation of let-7a1 by both mutant strains without significant differences compared to expression following wild-type infection (Figure 1).

There was no significant difference in expression of miR-146b, miR-16, let-7a1 and miR-145 between the Δ*hly* and Δ*inl*AB strains (Figure 1).

### 3.2. Wild-Type and LLO-Deficient Bacteria Induce miR-155, While the ΔinlAB Mutant Strain Suppresses miR-155 Expression

miR-155 is one of the best characterized miRNAs and is involved in innate immune response to a variety of pathogens, including but not limited to *H. pylori*, *P. syringae* and *Salmonella*. We show that *L. monocytogenes* induces strong miR-155 expression in Caco-2 cells (Figure 1). Strikingly, infection with Δ*hly* also provoked a comparable induction of miR-155. In contrast, Δ*inl*AB not only lacked the ability to induce of miR-155, but significantly downregulated miR-155 compared to wild-type infection and control (Figure 1).

### 3.3. Purified LLO Induces the Expression of miR-146b, miR-16 and miR-155 in Caco-2 Cells

**Figure 2:**
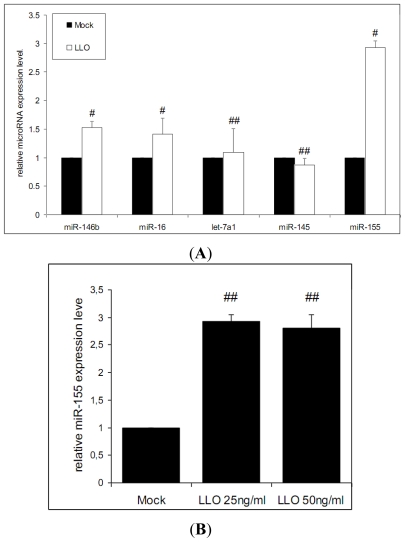
Deregulation of miRNAs following incubation with LLO. (**A**) The miRNA profile obtained from Caco-2 cells 1 h post infection for *L. monocytogenes* EGD-e wild-type was compared to Caco-2 cells treated with purified listeriolysin (LLO) for 1 h; # significant difference compared to control (*p*-value < 0.05), (**B**) miR-155 expression following incubation of Caco-2 cells with 25 ng/mL and 50 ng/mL LLO. Error bars indicate standard deviations. ## significant difference compared to control (*p*-value < 0.05), but no difference between different LLO concentrations (*p* > 0.05).

In a further step, we sought to investigate the regulation of the above studied miRNAs after incubation with purified LLO. Expression of three miRNAs, miR-146b, miR-16 and miR-155 was significantly increased in infected cells compared to non-infected controls (Figure 2A).

Strikingly, miR-146b displayed an inverted expression pattern compared to Δ*hly* infection indicating that expression of miR-146b is directly connected to the presence of LLO. While unchanged after Δ*hly* infection compared to control, miR-16 is seen upregulated after LLO incubation emphasizing the importance of this effector protein in miRNA regulation induced by *L. monocytogenes*. In contrast, induction of miR-155 expression was comparable in both settings, following infection with Δ*hly* strains as well as LLO incubation. To quantify the effect of higher doses of LLO on the magnitude of miR-155 induction we used a higher toxin dose. We observed no significant changes in miR-155 expression between both LLO toxin concentrations (Figure 2B).

In contrast to the changes seen in miR-155 expression, miR-145 and let-7a1 expression showed no significant deregulation in Caco-2 cells incubated with LLO (Figure 2A).

### 3.4. Deregulation of mRNAs That Are Targeted by miRNAs

**Figure 3:**
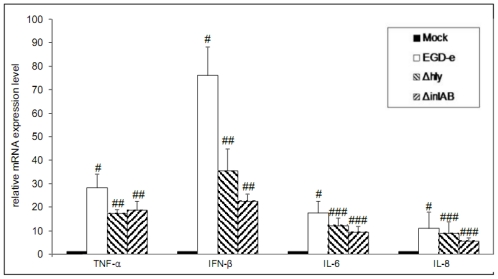
Conformation of immune response target genes by real time PCR analysis. Real time PCR analysis of immune response target genes was performed from uninfected Caco-2 cells compared to infected Caco-2 cells at 1 h post infection for *L. monocytogenes* EGD-e wild-type, Δ*hly* and Δ*inl*AB. Error bars indicate standard deviations. # significant difference compared to control (*p*-value < 0.05). ## significant difference compared with wild-type infection (*p*-value < 0.05), ### no significant difference compared with wild-type infection (*p*-value > 0.05).

To estimate the correlation between miRNA deregulation and downstream effects on target mRNA of particular miRNAs we measured mRNA expression levels of important targets of these miRNAs. These include major inflammatory cytokines and interleukins such as IL-6, IL-8, TNF-α, IFN-β (Figure 3 and Table 1).

In concordance with miRNA deregulation, there are significant changes of target mRNA levels in Caco-2 cells infected with wild-type and Δ*hly* or Δ*inl*AB mutant strains compared to control cells.

## 4. Discussion

In this study we demonstrate for the first time that *L. monocytogenes* mediates differential deregulation of miRNAs in the human epithelial cell line Caco-2. Using wild-type bacteria, two isogenic mutants Δ*hly* and Δ*inl*AB, and purified toxin we show that listeriolysin and internalins are involved in miRNA expression and regulation of the putative target transcripts. miRNA microarrays were used to screen and select a subset of miRNA candidates that were significantly deregulated and have biologically validated roles in host response to external stimuli. These miRNAs, including miR- 16, miR-145, mir146, miR-155 and let-7a1 were further investigated.

miR-16 is required for the rapid degradation of inflammatory mediators that contain AU-rich sequences, such as TNF-α, IL-6 and IL-8. Interestingly, miR-16 was previously reported to be upregulated in NIH 3T3 cells infected with murine gammaherpesvirus 68, a virus closely related to Epstein-Barr virus (EBV) and Kaposi’s sarcoma associated herpesvirus (KSHV) [21]. Activation of miR-16 gene was also observed in cholangiocytes in a p65-independent manner by *Cryptosporidium parvum*, a protozoan parasite that infects the gastrointestinal epithelium [22]. We observed a significant upregulation of miR-16 by wild-type bacteria and purified LLO, while absence of *hly* and *inl*AB resulted in significantly decreased expression of miR-16. Other studies have shown that miR-16 expression is stable among a variety of cell lines and expression is not altered by a variety of immune modulators. The observed toxin mediated induction of miR-16 and subsequent targeting of inflammatory mediators may therefore represent a targeted miRNA mediated mechanism of immunmodulation triggered by *L. monocytogenes* rather than an unspecific host cell response to infection [17,23,24].

miRNA expression profiling in human macrophages has shown that miR-146 and miR-155 are endotoxin-responsive genes that are involved in several immune and inflammatory pathways [25,26]. A recent study revealed that miR-146b upregulation leads to inhibition of *H. pylori* induced inflammatory response in human gastric epithelial cells. miR-146b was shown to inhibit IL-8 expression, possibly through interleukin-1 receptor-associated kinase 1 (IRAK1) and TNF receptor-associated factor 6 (TRAF6), two major adaptor molecules in TLR receptor signaling and NF-kB activation [27]. Thus miR-146b is a potent target to aim in order to manipulate host response. We show that miR-146b is mainly induced in a LLO-dependent manner during infection with *L. monocytogenes* and emphasize the central role of LLO the regulation of host miRNA. Caco-2 cells express TLR2 and TLR4 [28], two cell surface receptors that are targeted by listerial virulence factors including LLO. Thus, we suggest that *Listeria* induced miR-146b induction and subsequent target gene interaction may be triggered by LLO via a TLR-mediated pathway.

miR-155 has an established regulatory role in several pathways of innate and adaptive immune response [26]. Our results show that wild-type bacteria and purified LLO at two different doses induce miR-155 expression to a similar extent. However, upregulation of miR-155 also occurs following incubation with the LLO deficient mutant strain indicating that this induction is also triggered through a vacuole-dependent pathway. This process is possibly mediated by MyD88, since vacuolar signaling and subsequent expression regulation in listerial infection is entirely dependent on this adaptor molecule [29]. MyD88 also integrates TLR-signaling triggered by extracellular stimuli, such as LLO incubation. We conclude that miR-155 induction may be triggered through both LLO-dependent and an LLO-independent vacuolar mediated pathway. Both routes may merge in a common pathway that results in a comparable miR-155 induction as observed in this study.

Interestingly, the expression of miR-155 was strongly reduced following infection with Δ*inl*AB compared to wild-type bacteria or Δ*hly*. Thus, we suggest a new functional role for internalins in the regulation of miR-155 that subsequently results in increased degradation of the pro-inflammatory response mediated by TNF-α.

A recent study investigated the role of miR-145 in the inflammatory response in human colonic tissue of patients with ulcerative colitis [30]. miR-145 was strongly upregulated in inflamed colon segments of affected subjects who are at increased risk to develop colon cancer. A further study demonstrated that blocking miR-145 led to a strong anti-inflammatory response and reduced airway hyper responsiveness [15]. Thus, downregulation of miR-145 by *L. monocytogenes* as observed in this study may serve as a further mechanism of diminishing host immune response and facilitate survival of the pathogen. Furthermore, miR-145 was predicted to target IFN-β [18], a type I interferon that exhibits inflammatory and anti-inflammatory effects upon infection with *L. monocytogenes*. In line with miR-145 downregulation, IFN-β was strongly upregulated upon infection of Caco-2 cells indicating a possible contribution of miR-145 in its regulation, although it did not reach statistical significance.

Previous reports implicated miR-145 in the release of intestinal mucus components such as mucin (e.g., MUC1 or MUC2) that mediate an exocytosis mechanism leading to decreased uptake of *L. monocytogenes* into epithelial cells. *L. monocytogenes* was shown to counteract this mechanism via binding MUC2 by InlB, InlC and InlJ [31]. It is known that miR-145 controls the suppression of MUC1 causing a reduction of β-catenin, as well as the oncogenic cadherin 11 [32]. Thus, downregulation of miR-145 by the host cell results in decreased bacterial uptake. Overall miR-145 has a complex role in response to infection with *L. monocytogenes* and warrants further study.

Recently, downregulation of let-7 family members was identified as control major regulators of inflammation, including IL-6 and IL-10 in macrophages and HeLa cells upon infection with *Salmonella* [5]. We observed a similar regulation in Caco-2 cells following *Listeria* infection suggesting an analogous role of this host miRNA in Gram-positive and Gram-negative pathogens.

## 5. Conclusion

The results presented in this study contribute to our understanding of the host miRNA response induced by *L. monocytogenes* in intestinal epithelial cells. We show that (i) *L. monocytogenes* induces significant deregulation of miRNAs; (ii) major virulence determinants such as listeriolysin and internalins are involved in the regulation of a miRNA repertoire; and (iii) miRNAs interference may contribute to the post-transcriptional regulation of genes involved in the immune response to Gram-positive bacteria. Further studies are required to understand the mechanistic aspects of miRNA-mRNA interactions in the context of infections with Gram-positive pathogens. miRNAs may further expand our view on the role of non-coding RNAs as “effector-RNAs” within the eukaryotic host and represent a new target in the development of anti-microbial drugs.

## Supplementary Information



## Figures and Tables

**Table 1:** Comparison of fold changes of candidate miRNAs that were identified using miRNA microarrays and validated by qRT-PCR. and target mRNAs of each miRNA. The fold changes were display the relative miRNA expression in infected Caco-2 cells 1 h following infection with *L. monocytogenes* and control cells.

microRNA	FC microarray	FC qRT-PCR	target mRNA	Reference
miR-146b	1.43	1.28	IL-8, IL-6	[16]
miR-16	0.64	1.65	TNF-α, IL-6, IL-8	[17]
let-7a1	0.63	0.72	IL-10, IL-6	[5]
miR-145	0.39	0.39	IFN-β	[18]
miR-155	1.783	2.92	TNF-α, IFN-β	[19,20]

## References

[1] Ambros V. (2004). The functions of animal microRNAs. Nature.

[2] Huang Y., Shen X.J., Zou Q., Wang S.P., Tang S.M., Zhang G.Z. (2011). Biological functions of microRNAs: A review. J. Physiol. Biochem.

[3] Cullen B.R. (2011). Viruses and microRNAs: RISCy interactions with serious consequences. Genes Dev.

[4] Oertli M., Engler D.B., Kohler E., Koch M., Meyer T.F., Muller A. (2011). MicroRNA-155 is essential for the T cell-mediated control of *Helicobacter pylori* infection and for the induction of chronic Gastritis and Colitis. J. Immunol.

[5] Schulte L.N., Eulalio A., Mollenkopf H.J., Reinhardt R., Vogel J. (2011). Analysis of the host microRNA response to *Salmonella* uncovers the control of major cytokines by the let-7 family. EMBO J.

[6] Russo A., Potenza N. (2011). Antiviral effects of human microRNAs and conservation of their target sites. FEBS Lett.

[7] Plaisance-Bonstaff K., Renne R. (2011). Viral miRNAs. Methods Mol. Biol.

[8] Navarro L., Jay F., Nomura K., He S.Y., Voinnet O. (2008). Suppression of the microRNA pathway by bacterial effector proteins. Science.

[9] Rao J.R., Nelson D., Moore J.E., Millar B.C., Goldsmith C.E., Rendall J., Elborn J.S. (2010). Non-coding small (micro) RNAs of *Pseudomonas aeruginosa* isolated from clinical isolates from adult patients with cystic fibrosis. Br. J. Biomed. Sci.

[10] Xiao B., Liu Z., Li B.S., Tang B., Li W., Guo G., Shi Y., Wang F., Wu Y., Tong W.D. (2009). Induction of microRNA-155 during *Helicobacter pylori* infection and its negative regulatory role in the inflammatory response. J. Infect. Dis.

[11] Glaser P., Frangeul L., Buchrieser C., Rusniok C., Amend A., Baquero F., Berche P., Bloecker H., Brandt P., Chakraborty T. (2001). Comparative genomics of *Listeria* species. Science.

[12] Guzman C.A., Rohde M., Chakraborty T., Domann E., Hudel M., Wehland J., Timmis K.N. (1995). Interaction of *Listeria monocytogenes* with mouse dendritic cells. Infect. Immun.

[13] Parida S.K., Domann E., Rohde M., Muller S., Darji A., Hain T., Wehland J., Chakraborty T. (1998). Internalin B is essential for adhesion and mediates the invasion of *Listeria monocytogenes* into human endothelial cells. Mol. Microbiol.

[14] Rose F., Zeller S.A., Chakraborty T., Domann E., Machleidt T., Kronke M., Seeger W., Grimminger F., Sibelius U. (2001). Human endothelial cell activation and mediator release in response to *Listeria monocytogenes* virulence factors. Infect. Immun.

[15] Collison A., Mattes J., Plank M., Foster P.S. (2011). Inhibition of house dust mite-induced allergic airways disease by antagonism of microRNA-145 is comparable to glucocorticoid treatment. J. Allergy Clin. Immunol.

[16] Bhaumik D., Scott G.K., Schokrpur S., Patil C.K., Orjalo A.V., Rodier F., Lithgow G.J., Campisi J. (2009). MicroRNAs miR-146a/b negatively modulate the senescence-associated inflammatory mediators IL-6 and IL-8. Aging (Albany NY).

[17] Jing Q., Huang S., Guth S., Zarubin T., Motoyama A., Chen J., Di Padova F., Lin S.C., Gram H., Han J. (2005). Involvement of microRNA in AU-rich element-mediated mRNA instability. Cell.

[18] Witwer K.W., Sisk J.M., Gama L., Clements J.E. (2010). MicroRNA regulation of IFN-beta protein expression: Rapid and sensitive modulation of the innate immune response. J. Immunol.

[19] Tili E., Michaille J.J., Cimino A., Costinean S., Dumitru C.D., Adair B., Fabbri M., Alder H., Liu C.G., Calin G.A. (2007). Modulation of miR-155 and miR-125b levels following lipopolysaccharide/TNF-alpha stimulation and their possible roles in regulating the response to endotoxin shock. J. Immunol.

[20] O’Connell R.M., Taganov K.D., Boldin M.P., Cheng G., Baltimore D. (2007). MicroRNA-155 is induced during the macrophage inflammatory response. Proc. Natl. Acad. Sci. USA.

[21] Zhu J.Y., Strehle M., Frohn A., Kremmer E., Hofig K.P., Meister G., Adler H. (2010). Identification and analysis of expression of novel microRNAs of murine gammaherpesvirus 68. J. Virol.

[22] Zhou R., Hu G., Liu J., Gong A.Y., Drescher K.M., Chen X.M. (2009). NF-kappaB p65-dependent transactivation of miRNA genes following *Cryptosporidium parvum* infection stimulates epithelial cell immune responses. PLoS Pathog.

[23] Landgraf P., Rusu M., Sheridan R., Sewer A., Iovino N., Aravin A., Pfeffer S., Rice A., Kamphorst A.O., Landthaler M. (2007). A mammalian microRNA expression atlas based on small RNA library sequencing. Cell.

[24] Linsley P.S., Schelter J., Burchard J., Kibukawa M., Martin M.M., Bartz S.R., Johnson J.M., Cummins J.M., Raymond C.K., Dai H. (2007). Transcripts targeted by the microRNA-16 family cooperatively regulate cell cycle progression. Mol. Cell Biol.

[25] Taganov K.D., Boldin M.P., Chang K.J., Baltimore D. (2006). NF-kappaB-dependent induction of microRNA miR-146, an inhibitor targeted to signaling proteins of innate immune responses. Proc. Natl. Acad. Sci. USA.

[26] Quinn S.R., O’Neill L.A. (2011). A trio of microRNAs that control Toll-like receptor signalling. Int. Immunol.

[27] Liu Z., Xiao B., Tang B., Li B., Li N., Zhu E., Guo G., Gu J., Zhuang Y., Liu X. (2010). Up-regulated microRNA-146a negatively modulate Helicobacter pylori-induced inflammatory response in human gastric epithelial cells. Microbes Infect.

[28] Vora P., Youdim A., Thomas L.S., Fukata M., Tesfay S.Y., Lukasek K., Michelsen K.S., Wada A., Hirayama T., Arditi M. (2004). Beta-defensin-2 expression is regulated by TLR signaling in intestinal epithelial cells. J. Immunol.

[29] Leber J.H., Crimmins G.T., Raghavan S., Meyer-Morse N.P., Cox J.S., Portnoy D.A. (2008). Distinct TLR- and NLR-mediated transcriptional responses to an intracellular pathogen. PLoS Pathog.

[30] Pekow J.R., Dougherty U., Mustafi R., Zhu H., Kocherginsky M., Rubin D.T., Hanauer S.B., Hart J., Chang E.B., Fichera A. (2012). miR-143 and miR-145 are downregulated in ulcerative colitis: Putative regulators of inflammation and protooncogenes. Inflamm. Bowel Dis.

[31] Linden S.K., Bierne H., Sabet C., Png C.W., Florin T.H., McGuckin M.A., Cossart P. (2008). *Listeria monocytogenes* internalins bind to the human intestinal mucin MUC2. Arch. Microbiol.

[32] Sachdeva M., Mo Y.Y. (2010). MicroRNA-145 suppresses cell invasion and metastasis by directly targeting mucin 1. Cancer Res.

