# Supplementary material for: microRNA Response to Listeria monocytogenes Infection in Epithelial Cells
Source: Int J Mol Sci. 2012 Jan 20;13(1):1173–85. doi: 10.3390/ijms13011173 (PMC3269745; doi:10.3390/ijms13011173)
Supplement: Supplementary file 1 [file ijms-13-01173-s001.pdf]

Supplementary Information

Additional nucleotides are bound on the 5' end of each capture oligonucleotide necessary for enzymatic extension in the labelling procedure. The probes are synthesized with intra-array replicates to increase the statistical confidence and to compensate for potential positional effects. As a result the raw data files contain a total of 4 replicates for each miRNA with a total of 6456 data points. The intensities of blank probes which consist only of one single “T” nucleotide are used for background corrections. Spike-in controls for the labelling efficiency are also present. In order to control for the hybridization process as well as positioning features, additional hybridization controls are added to the array template. Blank, labelling control and hybridization control probes are not included in the data analysis.

The Geniom biochips were synthesized with the Geniom One instrument using febit's standard short-mer kit for oligonucleotide synthesis. The biochip was manufactured exactly to precise specifiations and achieved the standard quality criteria.

The quality control of eukaryotic total RNA was done with the Agilent 2100 Bioanalyzer, using the RNA 6000 Nano Kit in accordance with the manufacturer's instructions. Within the resulting electropherogram, high quality RNA is characterized by two distinct bands, representing the 18 and 28S rRNA, respectively.

For each array the RNA was suspended in febit's proprietary miRNA Hybridization Buffer (25 µL per array). Hybridization was done automatically for 16 h at 42 °C using the GeniomRTr-Analyzer.

After stringent washing and following the labelling procedure, microfluidic-based primer extension assay was applied. This assay utilizes the bound miRNAs as a primer for an enzymatic elongation with labelled nucleotides. Further elongation was done with Klenow Fragment and biotinylated nucleotides at 37 °C for 15 min. Finally, the biochip was washed automatically.

For maximum sensitivity, febit biotin and its detection with streptavidin-phycoerythrin (SAPE) was used, in combination with febit's consecutive signal enhancement (CSE) procedure. The feature recognition (using Cy3 filter set) and signal calculation were done automatically within milliseconds. The Geniom technology showed accurate detection of miRNA profiles. There was no photo bleaching thus enabling repeated measurements and multiple detection of each biochip.

Figure S1. Correlation between fold changes of investigated miRNAs measured by RT-PCR and microRNA microarrays. The graph shows log2 fold changes of miR-146b, miR-145, miR-155 and let-7a1 in infected Caco-2 cells. Thr outlier value of miR-16 measured by microarray was excluded from this correlation. R^2 = 0.69 when including miR-16. Overall, there is a strong correlation between fold changes measured by both independent techniques.

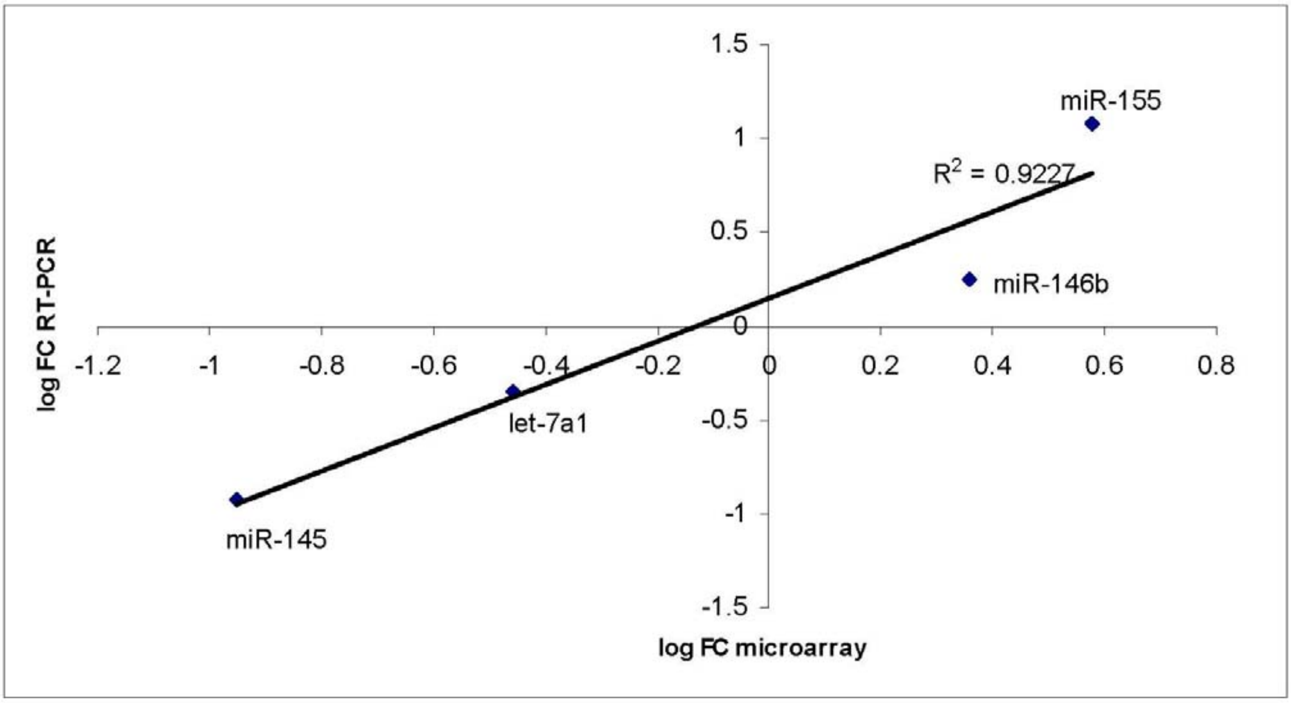

© 2012 by the authors; licensee MDPI, Basel, Switzerland. This article is an open access article distributed under the terms and conditions of the Creative Commons Attribution license (http://creativecommons.org/licenses/by/3.0/).
